# The Diagnostic Value of Ultrasound-Based Deep Learning in Differentiating Parotid Gland Tumors

**DOI:** 10.1155/2022/8192999

**Published:** 2022-05-12

**Authors:** Yaoqin Wang, Wenting Xie, Shixin Huang, Ming Feng, Xiaohui Ke, Zhaoming Zhong, Lina Tang

**Affiliations:** ^1^Department of Ultrasound, Fujian Medical University Cancer Hospital, Fujian Cancer Hospital, Fuzhou, 350014 Fujian Province, China; ^2^College of Electronic and Information Engineering, Tongji University, Shanghai 201804, China

## Abstract

**Objectives:**

Evidence suggests that about 80% of all salivary gland tumors involve the parotid glands, with approximately 20% of parotid gland tumors (PGTs) being malignant. Discriminating benign and malignant parotid gland lesions preoperatively is vital for selecting the appropriate treatment strategy. This study explored the diagnostic performance of deep learning system for discriminating benign and malignant PGTs in ultrasonography images and compared it with radiologists*. Methods*. A total of 251 consecutive patients with surgical resection and proven parotid gland malignant or benign tumors who underwent preoperative ultrasound examinations were enrolled in this study between January 2014 and November 2020. Next, we compared the diagnostic accuracy of deep learning methods (ViT-B\16, EfficientNetB3, DenseNet121, and ResNet50) and radiologists in parotid gland tumor. In addition, the area under the curve (AUC), specificity, sensitivity, positive predictive value, and negative predictive value were calculated.

**Results:**

Among the 251 patients, 176/251 were the training set, whereas 75/251 were the validation set. Results showed that 74/251 patients had malignant tumor. Deep learning models achieved good performance in differentiating benign from malignant tumors, with the diagnostic accuracy and AUCs of ViT-B\16, EfficientNetB3, DenseNet121, and ResNet50 model being 81% and 0.81, 80% and 0.82, 77% and 0.81, and 79% and 0.80, respectively. On the other hand, the diagnostic accuracy and AUCs of radiologists were 77%-81% and 0.68-0.75, respectively. It was evident that the diagnostic accuracy of deep learning methods was higher than that of inexperienced radiologists, but there was no significant difference between deep learning methods and experienced radiologists.

**Conclusions:**

This study shows that the deep learning system can be used for diagnosing parotid tumors. The findings also suggest that the deep learning system may improve the diagnosis performance of inexperienced radiologists.

## 1. Introduction

Parotid glands are important exocrine organs and the most frequent site of salivary gland tumors. Evidence suggests that 80% of all salivary gland neoplasms involve the parotid gland, with approximately 80% of parotid gland tumors (PGT) being benign and 20% being malignant [1]. Pleomorphic adenoma and Warthin tumor are the most common types of benign tumors, whereas mucoepidermoid carcinoma and adenoid cystic carcinoma are the most common malignant tumors in the parotid gland [2]. Most PGTs often have no specific manifestation and are characterized by a painless palpable mass on one or both sides. Currently, surgical resection is the main treatment strategy. However, discriminating benign and malignant parotid gland lesions preoperatively is critical for selecting the best treatment strategy [3]. A presumably benign PGT can be treated by local excision or a lateral parotidectomy. On the other hand, malignant lesions require more radical surgery approaches, such as an expanded scope of resection combined with lymph node dissection, which might result in more complication and invasive harm for the patients.

Classification of PGTs is hampered by the diverse variations in their histopathology. Presurgical determination of benign or malignant PGTs involves two main methods: fine needle aspiration cytology (FNAC) [4] and imaging modalities [5]. Although preoperative FNAC examination can improve the diagnostic accuracy, its use in the diagnosis of PGT is controversial. One study reported a sensitivity and negative predictive value (NPV) of 66.7% and 81.6% [4], respectively, whereas Dhanani et al. [6] reported a sensitivity of 88.9% in the diagnosis of parotid gland lesions. Moreover, FNAC is an invasive intervention method that has malignant tumor cell seeding risk that leads to tumor dissemination and recurrence. Despite computed tomography (CT) or magnetic resonance imaging (MRI) having superior qualities in distinguishing parotid gland lesions [7], they also have some limitations, including ionizing radiation, patients have contraindications for internal ferromagnetic devices, high monetary cost, and takes a long time.

It is worth noting that PGTs are easily accessible because they are commonly located in the superficial lobe and thus are very good for ultrasonography (US) detection [8]. US is a well-accepted imaging technique for the diagnosis of most PGTs because it is sensitive, noninvasive, no ionizing radiation, and inexpensive. US can depict the location, size, and characteristics of PGT, including the shape, margin, echogenicity, architecture, posterior echo enhancement, cystic component, calcification, and vasculature. Benign tumors always present with well-defined margins and regularities, whereas malignancies show irregular, heterogeneous, and high vascularization patterns [9]. Although grayscale, color Doppler flow, and shear wave elastography (SWE) can show PGT sonographic features, it is challenging to distinguish between benign and malignant tumors because these ultrasound features overlap broadly within malignant and benign nodes. A previous study reported that the sensitivity and specificity of B-mode US for differentiating malignant from benign nodules were 38.9% and 90.1%, respectively [10]. Therefore, this calls for development of more reliable methods for differentiating malignant from benign nodules, with the overarching goal of increasing the PGT differentiation diagnosis rate in US. In this study, we aimed at exploring whether combining US with an additional application can achieve satisfactory results without using invasive methods.

In recent years, deep learning (DL) is a technique that involves many layers; particularly convolution neural networks (CNN) and transformer are very applied to medical image segmentation and classification [11]. Several studies have reported that the diagnostic ability of deep learning systems for medical image diagnosis has achieved comparable results in various fields, including thyroid cancer [12], breast cancer [13], and liver tumor [14]. In addition, a previous study found that a deep learning system had a high diagnostic ability for Sjögren's syndrome using ultrasonography images [15]. However, to the best of our knowledge, few studies have explored deep learning methods on US data for distinction of PGTs. This study explored the diagnostic performance of deep learning for differentiating begin and malignant parotid gland tumors and compared the findings with results reported by radiologists.

## 2. Methods and Materials

### 2.1. Patients

This retrospective study was approved by the ethics committee of the Fujian Cancer Hospital (K2021-103-01). Written consent was obtained before surgery for each patient. The study recruited all patients with surgical resection and proven parotid gland malignant or benign tumors who underwent preoperative ultrasound examinations at a tertiary medical center between January 2014 and November 2020. Histopathological findings from the surgical samples were used as the gold standard in all cases. The inclusion criteria were as follows: (1) US examination performed in our hospital, (2) optimal quality of US images, (3) masses that were located in the superficial lobe, and (4) lesions diagnosed based on US findings. The exclusion criteria were as follows: (1) patients with a history of parotid gland surgery, (2) small lesions with<0.5 cm maximal diameter, and (3) lesions proven to be inflammatory.

### 2.2. Ultrasound Protocol

A total of 251 consecutive patients who meet the inclusion and exclusion criteria were included in the study. Participants comprised of 153 men and 98 women, with a median age of 54 years (range, 12-82 years). A detailed ultrasound scan of the head and neck region was carried out before surgery. US was performed for all patients using a 5-12 MHz linear-array transducer, such as iU22, Philips Medical Systems, GE E11, Supersionic Aixlporer. Conventional US was used to show the following characteristics of lesions: maximum diameter, margin (well/poorly defined), shape (regular/irregular), echogenicity (homogeneous/heterogeneous), posterior acoustic enhancement (absent/present), cystic component (absent/present), calcification (absent/present), and vasculature (grade 0/I/II/III). B-mode images that contained lesions with maximal diameter or plane with suspicion of malignant feature were extracted for deep learning analysis. Next, all images were independently reviewed by three ultrasound radiologists (YQW with more than 15 years' experience of US examination, SXH with more than eight years' experience of US examination, and ZMZ with less than five years' experience of US examination) who were blinded to the final histopathology result.

According to Adler's method for evaluating the vascular distributions of lesions, grade 0 is no determined vascularity in lesions; grade I is minimal blood flow, 1 ~ 2 point-like or thin rod vessels in the lesion, and the thin rod vessels do not exceed 1/2 of the diameter of the lesion; grade II is moderate blood flow and 3 ~ 4 punctate vessels or one important vessel whose length can be close to or exceed the diameter of the lesion; and grade III is rich blood flow, and more than five punctate vessels or two longer vessels were observed [16].

### 2.3. Data Preprocessing

**Figure 1:**
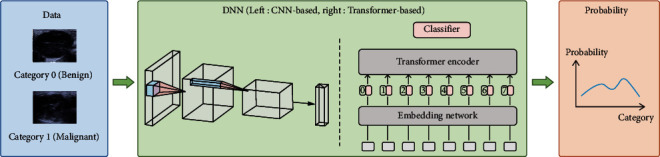
Development and validation of deep learning system for diagnosis of parotid glands lesions. DNN: deep neural network.

The flowchart was shown in Figure 1.

The datasets were split into training, validation, and testing sets through random partitioning, each consisting of 50%, 20%, and 30% of the total data. The training and validation groups consisted of 124 benign and 52 malignant patients, whereas the testing group consisted of 53 benign and 22 malignant patients. To obtain reliable results, the following data augmentation methods were applied during the training stage:

1. *Random Flipping*. A random flip with a probability of 0.5
2. *Random Rotating*. A random rotate of at most 10 degrees with a probability of 0.5
3. *Gaussian Blurring*. A square Gaussian kernel with 5 × 5 was used, with a probability of 0.5
4. *Random Lighting*. A random light of at most 20% with a probability of 0.5

Specifically, the augment data is only randomly generated during the training stage, and the validation and testing stages do not include the data augmentation step.

### 2.4. Diagnostic Performance of the Deep Learning System

Two common types of deep learning models were applied for classification: convolutional-based and transformer-based methods, with a total of four models. The convolutional-based methods include ResNet50 (https://arxiv.org/abs/1512.03385), DenseNet121 (https://arxiv.org/abs/1608.06993), and EfficientNetB3 (https://arxiv.org/abs/1905.11946). On the other hand, the transformer-based method was ViT-B\16 (https://arxiv.org/abs/2010.11929). Specifically, ResNet proposed a residual structure that alleviates the gradient dispersion problem in a deep neural network, DenseNet can reduce the number of network parameters through feature reuse, and EfficientNet expands on depth, width, and resolution in the network to achieve better efficiency and accuracy. Moreover, ViT introduces the transformer model (https://arxiv.org/abs/1706.03762) in natural language processing (NLP) into computer vision, thereby achieving better performance than convolutional-based methods.

For the practical implementation, we employed the PyTorch framework (https://pytorch.org/) and trained the models until convergence on a single NVIDIA 2080Ti GPU (NVIDIA Corp., Santa Clara, CA, USA). The optimal model was determined based on the metrics on the validation set, and the network was optimized using stochastic gradient descent (SGD) with a batch size of 4. The learning rate was initialized as 1*e*-3. Meanwhile, we employed cross-entropy loss as a loss function:

(1) $$\begin{array}{c} {\text{Loss}}_{CE}=-\frac{1}{n}\overset{n}{\underset{i=1}{\sum }}\left(y_{i}\cdot \log\left(p_{i}\right)+\left(1-y_{i}\right)\cdot \log\left(1-p_{i}\right)\right), \end{array}$$

where *n* is the total sample number, *y*_*i*_ is the ground truths, and *p*_*i*_ is the prediction probability.

Seven metrics were used to evaluate the performance, including accuracy, area under curve (AUC), f1 score, sensitivity, specificity, positive predictive value (PPV), and negative predictive value (NPV). Specifically, the f1 score can balance the precision and recall and is suitable for evaluating imbalanced datasets. In addition, the accuracy indicates the overall agreement between predictions and labels.

### 2.5. Statistical Analysis

All statistical analyses were performed using SPSS version 22.0 (IBM Corp.; Chicago, IL, USA). All parotid gland lesions were divided into malignant and benign groups. Kolmogorov-Smirnov test was used to determine normal distribution in groups. Data that were suitable for normal distribution were recorded as “mean ± standard deviation.” Given that age and lesion size did not follow a normal distribution, Mann–Whitney *U* tests were performed and recorded as “median (25th-75th percentile).” The Chi-square test was used to evaluate the categorical data, whereas Kendall's *W* test was applied to assess interobserver agreement among the three radiologists. Receiver operating characteristic (ROC) curves were generated to determine diagnostic performance. Finally, the specificity, sensitivity, PPV, and NPV of the models and radiologists in groups were calculated. *P* < 0.05 was considered statistically significant.

## 3. Results

### 3.1. Evaluation of Clinical Data and Ultrasound Characteristics

Table 1 shows the obtained US images depicting PGT for the training and validation sets that underwent imaging between January 2014 and November 2020. A total of 251 PGTs were included in this study. Among them, 176/251 (70.12%) were grouped in the training set, and 75/251 (29.88%) were grouped in the validation set. With regard to tumor classification, 93/251 (37.05%) had pleomorphic adenoma, 61/251 (24.30%) had Warthin's tumor, and 74/251 (29.48%) had malignant tumor.

All patients were divided into two groups: the benign group (177 patients, 153 males and 98 females; median age 54 years; median lesion size was 25 mm; 103 located on the left, and 74 located on the right) and malignant group (74 patients, 41 males and 33 females; median age 54 years; median lesion size was 28 mm; 42 located on the left, and 32 located on the right).  Table 2 shows the clinical data for the benign and malignant groups. Results showed that there was no significant difference between the two groups in age (*P* = 0.77), gender distribution (*P* = 0.31), lesion size (*P* = 0.06), and location (*P* = 0.94).

Ultrasound characteristics among the parotid gland benign and malignant tumor groups are shown in Table 2. Among the 177 parotid gland benign tumors, 173 (97.74%) showed well defined margins, 129 (72.88%) were regular in shape, 133 (75.14%) showed posterior echo enhancement, and 174 (98.30%) had no calcification. Among the 74 parotid gland malignant tumors, 64 (86.48%) were heterogeneous in echogenicity, 55 (74.32%) had irregular shape, 51 (68.92%) had absent posterior echo enhancement, and 32 (34.24%) exhibited grade III vascular pattern on CDFI. The results showed significant differences between the two groups with regard to margin definition, echogenicity, shape, posterior echo enhancement, calcification, and vascularization. On the other hand, there was no significant difference in the cystic component.

### 3.2. Performance of Deep Learning Models for Differentiating Benign from Malignant Tumors

**Figure 2:**
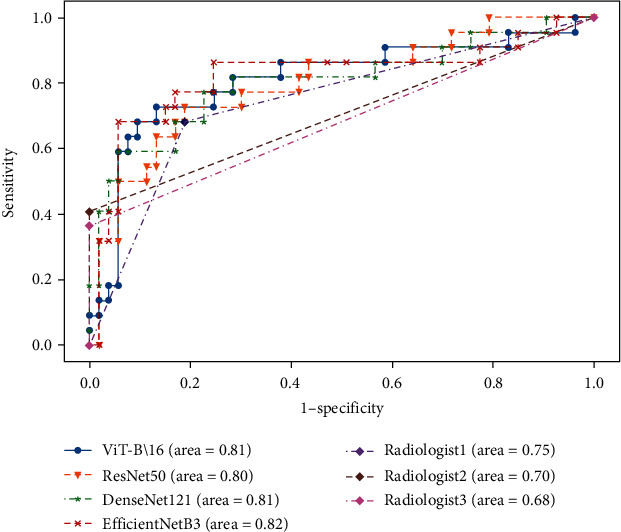
ROC curves (*N* = 75). The figure shows a comparison between the deep learning system and radiologists in diagnosing of PGTs.

Table 3 summarizes diagnostic performances of deep learning models for differentiating benign from malignant. Results indicated that the deep learning models achieved good performance in differentiating benign from malignant tumors, with diagnostic accuracy and AUCs of 81% and 0.81 for the ViT-B\16 model, 80% and 0.82 for the EfficientNetB3 model, 77% and 0.81 for the DenseNet121 model, 79% and 0.80 for the ResNet50 model, and 77% and 0.75 for experienced radiologist, respectively. The diagnostic accuracy and AUCs of inexperienced radiologists were 0.68 and 0.75, respectively. The ROC curves for model performance and the radiologists are shown in Figure 2.

## 4. Discussion

Evidence has shown that most PGTs are benign, with only 20% being malignant. However, distinguishing between benign and malignant parotid gland tumors is challenging. US is a low cost technique that is a well-accepted by patients. Therefore, numerous ultrasound modalities have been established for defining characteristics of lesions in efforts to assess the nature of PGT, including grey-scale US, color-Doppler US, superb microvascular imaging [17], elastography [18], and contrast-enhanced US. On US, benign parotid gland lesions typically present well-defined margins, homogeneous or inhomogeneous echotexture, and acoustic enhancement. On the other hand, high-grade malignant tumors usually present irregular, heterogeneous echotexture, and cervical lymph nodes spread, whereas low-grade malignant tumors may present as benign lesions. On Doppler US, malignant tumors and Warthin tumors have rich vascularization, whereas pleomorphic adenoma appears reduced vascularization. One study reported that there was appreciable overlap between the US features of benign tumors and that of malignant tumors within the histological heterogeneous [19]. Therefore, it is difficult to differentiate malignant and benign lesions based on B-mode and Doppler US.

According to the guidelines provided by several authors, we presume that poorly defined margins, heterogeneous or homogeneous structure, irregular, and high vascularity to be a possible standard for malignant tumors, whereas well defined, heterogeneous or homogeneous structure, regular, and posterior acoustic enhancement are likely criteria for begin tumors. Results obtained in this study showed that benign lesions had well-defined margins, with only 2.25% (4/177) having poorly defined margins. On the other hand, 39.18% (29/74) of the malignant lesions showed poorly defined margins, whereas 60.81% (45/74) exhibited well-defined margins, which is consistent with results reported by Bozzato et al. [20]. This may be attributed to the fact that low-grade malignant tumors appear well-defined, and only high-grade malignant tumors present poorly defined margins. Most malignant tumors present heterogeneous echogenicity (86.48%, 64/74) and irregular shape (74.3%, 55/74), whereas most benign lesions present a regular shape (72.88%, 29/177). Benign lesions could also show heterogeneous echogenicity, especially pleomorphic adenoma with cysts and calcification. In addition, 75.14% (133/177) of benign lesions exhibited posterior acoustic enhancement. Rzepakowska et al. [21] revealed that the increased vascularity pattern was the most reliable feature for the assessment of malignant lesions. This study found that most malignant lesions (63.51% (47/74)) show moderate or rich blood flow in lesions, which is consistent with Rzepakowska et al. [21]. However, all these US approaches can be routinely used in clinical practice, but with some limitations since US depends on the experience of the radiologist and is difficult for junior radiologists. Therefore, there is need to develop a new technique to objectively evaluate ultrasound images in clinical work.

This study also investigated the diagnosis performance of a novel method, a deep learning system that is based on a deep neural network, in characterization of parotid gland lesions. It should be noted that there are numerous published articles showing that deep learning can be useful for the diagnosis and management of various tumors [21]. There have also been several reports on characterizing PGTs using deep learning. For example, Matsuo et al. [22] reported that the deep learning method could discriminate benign and malignant PGTs in MRI images, with an AUC of 0.86. Gabelloni et al. [24] used magnetic resonance radiomics to discriminate PGTs, with results showing that radiomics analysis had a high diagnostic performance in pleomorphic adenomas and malignant tumors (sensitivity, specificity, and diagnostic accuracy of 0.66, 0.87, and 0.80, respectively). However, no study has explored whether deep learning can be applied in US images to differentiate benign from malignant tumors. The findings of this study showed that deep learning methods could improve performance in terms of the differential diagnosis of parotid gland benign and malignant tumors. Among the four deep learning models investigated in this study, the EfficientNetB3 model provided the best classification results, with the accuracy, sensitivity, and AUC being 80%, 77%, and 0.82. We found that deep learning software differentiated parotid gland lesions with good diagnostic accuracy (AUC = 0.82) and a high negative predictive value (NPV = 90%). Radiologists demonstrated comparable accuracy (AUC 0.68-0.75) at a lower NPV (79-86%). Although the diagnostic accuracy of deep learning was higher than that of inexperienced radiologists, there was no significant difference between deep learning systems and experienced radiologists. This suggests that the use of deep learning system analysis is more reliable than descriptive evaluation in diagnosing PGT in inexperienced radiologists. Additional, Santos et al. [25] have reported that contrast-enhanced computed tomography can evaluate the morphology, volume, and density of the parotid glands before and after chemoradiation therapy in head and neck cancer patients. This indicated that those changes in parotid glands may be also detected by the ultrasound-based deep learning system.

This study had several limitations. First, the number of cases was relatively small. However, the number of parotid gland malignant lesions included was 74 of 251 (29.4%), which is consistent with previous studies. Nevertheless, a large-scale sample may be required in further research to improve the performance. It should be noted that the occurrence of parotid gland malignancies in routine clinical work is relatively rare; thus, it seems difficult to increase the number of malignant cases. Second, the retrospective data might be a limitation if the examination is carried out with different ultrasound equipment and by different examiners. Therefore, prospective studies should be included in future research. Third, lesions located in the deep lobe have certain limitations. Notably, there were no deep lobe lesions cases in our study since US was not appropriate for these lesions.

## 5. Conclusion

In conclusion, B mode US evaluation of PGT may be difficult due to the appreciable overlap characteristics in benign tumors and malignancies. The use of a deep learning system had a promising diagnostic ability for differentiating a benign from a malignant PGT. This suggests that deep learning could be used for diagnostic support in the assessment of parotid gland lesions in inexperienced radiologists.

## Figures and Tables

**Table 1:** The histopathologic diagnosis of the parotid masses and data distribution of the training, validation, and testing sets.

Tumor type	Training and validation	Testing	Total
Benign	124	53	177
Pleomorphic adenoma	59	34	93
Warthin's tumor	48	13	61
Other benign tumors	17	6	23
Malignant	52	22	74
Mucoepidermoid carcinoma	8	6	14
Acinic cell carcinoma	8	4	12
Salivary duct carcinoma	7	2	9
Lymphoma	6	1	7
Metastases	5	4	9
Other malignant tumors	18	5	23
Total cases	176	75	251

**Table 2:** Clinical features and ultrasound characteristics among benign and malignant parotid gland tumors.

Features	Benign (*n* = 177)	Malignant (*n* = 74)	*P*
Age	54 (42-62)	54 (39.25-63)	0.77
Gender			0.31
Male	112	41	
Female	65	33	
Lesion size (mm)	25 (19-33)	28 (21.25-35.75)	0.06
Location			0.94
Left	103	42	
Right	74	32	
Margin definition			1.475*e*-14
Well defined	173	45	
Poorly defined	4	29	
Echogenicity			5.253*e*-06
Homogeneous	79	10	
Heterogeneous	98	64	
Shape			1.108*e*-11
Regular	129	19	
Irregular	48	55	
Posterior acoustic enhancement			1.366*e*-10
Absent	44	51	
Present	133	23	
Cystic component			0.71
Absent	137	55	
Present	40	19	
Calcification			8.121*e*-07
Absent	174	59	
Present	3	15	
Vascularization			2.031*e*-07
0	32	9	
I	64	18	
II	33	15	
III	48	32	

**Table 3:** Performance of four DNN models and radiologists according to validation set.

	ViT-B\16	EfficientNetB3	DenseNet121	ResNet50	Radiologist1	Radiologist2	Radiologist3
AUC	0.81	0.82	0.81	0.80	0.75	0.70	0.68
Accuracy	81 (61/75)	80 (60/75)	77 (58/75)	79 (59/75)	77 (58/75)	82 (62/75)	81 (61/75)
Sensitivity	68 (15/22)	77 (17/22)	64 (14/22)	73 (16/22)	68 (15/22)	41 (9/22)	36 (8/22)
Specificity	87 (46/53)	81 (43/53)	83 (44/53)	81 (43/53)	81 (43/53)	100 (53/53)	100 (53/53)
PPV	68 (15/22)	63 (17/27)	61 (14/23)	62 (16/26)	60 (15/25)	100 (4/4)	100 (3/3)
NPV	87 (46/53)	90 (43/48)	85 (44/52)	88 (43/49)	86 (43/50)	80 (53/66)	79 (53/67)
F1 score	68	69	62	67	67	58	53

DNN: deep neural network.

## Data Availability

The data used to support the findings of this study are included within the article.
